# Canine Cutaneous Leishmaniasis: Dissemination and Tissue Tropism of Genetically Distinct *Leishmania (Viannia) braziliensis* Populations

**DOI:** 10.1155/2013/982183

**Published:** 2013-06-12

**Authors:** Guilherme Marx de Oliveira, Maria de Fátima Madeira, Fernanda Santos Oliveira, Marize Quinhones Pires, Raquel da Silva Pacheco

**Affiliations:** ^1^Laboratório de Epidemiologia e Sistemática Molecular, Instituto Oswaldo Cruz, FIOCRUZ, Avenida Brasil No. 4365, Manguinhos, 21040-360 Rio de Janeiro, RJ, Brazil; ^2^Laboratório de Vigilância em Leishmanioses, Instituto de Pesquisa Clínica Evandro Chagas, FIOCRUZ, Avenida Brasil No. 4365, Manguinhos, 21040-360 Rio de Janeiro, RJ, Brazil

## Abstract

Little is known regarding the internal dissemination of initial cutaneous lesions and tissue tropism of *Leishmania (Viannia) braziliensis* populations in naturally infected dogs. The aim of this study was to investigate genetic polymorphisms of *L. (V.) braziliensis* populations in different anatomic sites of naturally infected dogs by using polymerase chain reaction (PCR) and low-stringency single specific primer-PCR (LSSP-PCR) techniques. The amplified products were analyzed by LSSP-PCR to investigate the genetic variability of the parasite populations present in different anatomical sites. Twenty-three out of the 52 samples gave PCR-positive results. The existence of *L. (V.) braziliensis* strains that remained restricted to cutaneous lesions and others showing characteristics of dissemination to internal organs and healthy skin was observed. LSSP-PCR and numerical analyses revealed that parasite populations that do not disseminate were genetically similar and belonged to a separate phenetic cluster. In contrast, populations that showed spreading to internal organs displayed a more polymorphic genetic profile. Despite the heterogeneity, *L. (V.) braziliensis* populations with identical genetic profiles were observed in popliteal and cervical lymph nodes of the same animal. Our results indicate that infection in dogs can be manifested by dissemination and tissue tropism of genetically distinct populations of *L. (V.) braziliensis*.

## 1. Introduction

Leishmaniasis is a protozoal disease caused by different species of the genus *Leishmania*, and it is transmitted by the bite of female phlebotomine insects of the genus *Lutzomyia *in the New World. Cutaneous leishmaniasis (CL) and visceral leishmaniasis (VL) are endemic in large areas of the tropics, subtropics, and the Mediterranean basin. Around 350 million people in 88 countries are at risk of contracting leishmaniasis, and this number is believed to be inaccurate due to the flaws in the case detection data used to estimate the disease prevalence in many endemic countries [1].

In the state of Rio de Janeiro, *Leishmania (Viannia) braziliensis* is the most prevalent species that is responsible for CL. Transmission mainly occurs in periurban areas, where the primitive rain forest vegetation is being replaced by disorganized human occupation. Adaptation of the vector *Lutzomyia intermedia* to the domiciliary and peridomiciliary environments, as well as the presence of infected humans, dogs, and horses, has been observed [2–4]. In the canine population, *L. (V.) braziliensis* infection is manifested by cutaneous lesions, which are usually located in the scrotum, ear, and muzzle, and these usually do not affect the general health of the animal.

Little is known regarding internal dissemination of an initial cutaneous lesion and tissue tropism of parasitic populations in naturally infected dogs. Although polymerase chain reaction (PCR) can be used to detect the presence of *L. (V.) braziliensis *DNA in blood samples from naturally infected dogs [5], the search for a more sensitive molecular technique capable of detecting the polymorphic features of the parasite is required.

The aim of the present study was to investigate the genetic polymorphism of *L. (V.) braziliensis* populations present in different anatomic sites of naturally infected dogs from the endemic areas of Rio de Janeiro, Brazil. The use of PCR and low-stringency single specific primer-PCR (LSSP-PCR) as an integrated approach has enabled the simultaneous diagnosis and genetic typing of specific populations of *L. (V.) braziliensis* in the canine CL.

## 2. Materials and Methods

Fifty-two clinical samples from 9 dogs were analyzed. The animals were referred to the Zoonosis Service, Evandro Chagas Clinical Research Institute, Oswaldo Cruz Foundation, with an indication for euthanasia according to the recommendations of the Brazilian Program for the Control of Leishmaniasis [6], after serological evaluations. All the animals proceeded from VL endemic areas in Rio de Janeiro, and they were euthanized by thiopental overdose before the clinical samples were collected. The samples consisted of biopsy fragments of skin lesions, healthy skin from the scapular region, spleen, liver, and cervical and popliteal lymph nodes. Infection in these animals was also confirmed by the isolation of *L. (V.) braziliensis* in fragments of cutaneous lesions. This study was approved and licensed by the Ethics Committee of Animal Users (CEUA-FIOCRUZ) under no. L-23/06.

### 2.1. DNA Isolation and Specific PCR Assays

For DNA isolation, the Genomic Prep Cells and Tissue DNA Isolation kit (Amersham Pharmacia) was used according to the protocol described by Oliveira et al. [7]. The parasite DNA was detected by PCR amplification using a pair of primers (B1: 5′-GGGGTTGGTGTAATATAGTGG-3′; B2: 5′-CTAATTGTGCACGGGGAGG-3′) that amplifies the variable region of kDNA minicircles of species of the subgenus *Viannia* belonging to *Leishmania braziliensis* complex [8]. The reaction was performed in a final volume of 25.0 *μ*L containing 3.0 *μ*L of sample DNA, 10 mM of Tris-HCl buffer solution, 1.5 mM of MgCl_2_, 0.2 mM of deoxynucleoside triphosphate (dNTPs), 10 pmol of each primer, and 2.5 U of Taq DNA polymerase. Amplification cycles were started at 95°C for 3 min, followed by 33 cycles at 94°C for 30 s, 60.5°C for 1 min, 72°C for 1 min, and a final extension at 72°C for 10 min. A positive control containing the kDNA (5 ng/*μ*L) of the reference strain *L. (V.) braziliensis* (MHOM/BR/1975/M2903) and a negative control containing all the components, except DNA, were used. Samples with negative results were reevaluated and subjected to the inhibition test. The amplified products were analyzed in 1.5% agarose gels, visualized under ultraviolet light, and photographed using a UVP GDS 7500 apparatus (Gel Documentation System).

### 2.2. Low-Stringency Single Specific Primer PCR Analysis

*L. (V.) braziliensis* kDNA 750-bp fragments generated by specific PCR were purified with the Wizard PCR Prep system (Promega, Madison, WI, USA) according to the manufacturer's instructions. LSSP-PCRs were performed by amplifying the purified DNA fragments with the B2 primer: 5′-CTAATTGTGCACGGGGAGG-3′. Reactions were carried out with 40 pmol of primer, 200 *μ*moL/L dNTPs, 10 mmol/L Tris-HCl (pH 8.6), 50 mmol/L KCl, 1.5 mmol/L MgCl_2_, 4 U of Taq polymerase, and 3 *μ*L (60 ng/*μ*L) of the purified 750-bp fragment (final volume, 25 *μ*L). Amplification was performed in a thermocycler as follows: 95°C for 5 min; 95°C for 1 min, 36°C for 30 s, and 72°C for 2 min (45 cycles); and final extension at 72°C for 10 min. Amplification products were analyzed on 1.8% agarose gels (High Resolution, Sigma) after ethidium bromide staining.

### 2.3. Numerical (Phenetic) Analysis

Bands ranging in size from 300 to 750 base pairs (bp) were selected for phenetic analysis. The LSSP-PCR profiles were compared using the simple matching (Sm) coefficient of similarity to determine the proportion of mismatched bands. The similarity matrix was transformed into a dendrogram by the unweighted pair group method arithmetical average (UPGMA) algorithm [9] using the NTSYS program (version 2.0; Exeter Software, Setauket, NY, USA).

## 3. Results

### 3.1. Clinical Condition and PCR Assays of the Animals under Study

Fifty-two samples from nine dogs from endemic areas of Rio de Janeiro, Brazil, were evaluated. The animals were seropositive for *Leishmania,* and indirect immunofluorescence antibody test (IFAT) showed that they had different serological titers. Cutaneous lesions were present mainly in the muzzle, ears, and scrotum. The IFAT titers, lesion localizations, and geographical origin of the animals are shown in Table 1. Specific PCR assays were performed in 52 clinical samples obtained from the animals under study. Twenty-three out of the 52 samples gave positive results after PCR amplifications. The 750-bp diagnostic bands specific to parasites of the *L. braziliensis* complex were detected in the biopsy fragments as follows: 100% (9/9) in cutaneous lesions, 30% (3/9) in healthy skin, 44.4% (4/9) in spleen, 22.2% (2/9) in liver, 42.9% (3/7) in popliteal, and 22.2% (2/9) in cervical lymph nodes. Two samples from the lymph nodes were lost during DNA extractions. Interestingly, it was noted that *L. (V.) braziliensis* strains remained restricted to the cutaneous lesions in the animals A3, A4, A5, and A6, while the others (animals A1, A2, A7, A8, and A9) showed characteristics of dissemination in the internal organs and in healthy skin (Table 2).

### 3.2. LSSP-PCR Assays and Phenetic Analyses

The PCR-positive products (750-bp bands) were subjected to LSSP-PCR. Different degrees of genetic polymorphisms were observed in the amplified variable regions of *L. (V.) braziliensis* kDNA minicircles. The reproducibility of the method was confirmed when identical electrophoretic profiles were observed in the assays on repeated analysis under the same conditions.

**Figure 1:**
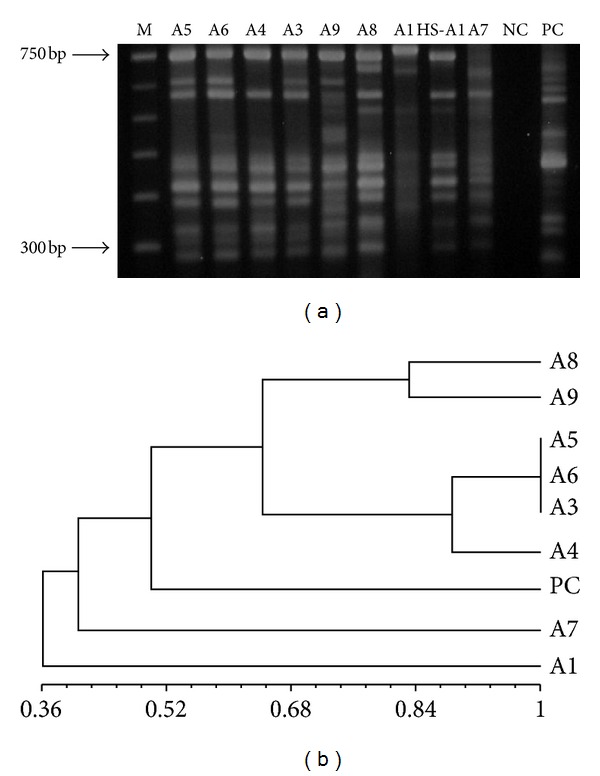
(a) Agarose gel electrophoresis showing representative genetic profiles generated by LSSP-PCR from parasites of cutaneous lesions of 8 animals (A8, A9, A5, A6, A3, A4, A7, and A1); HS-A1: health skin from animal A1. (b) Phenetic analysis using the Jaccard coefficient of similarity and UPGMA algorithm. M: 100 bp DNA marker; NC: negative control; PC: positive control (*L. (V.) braziliensis* reference strain MHOM/BR/1975/M2903).

**Figure 2:**
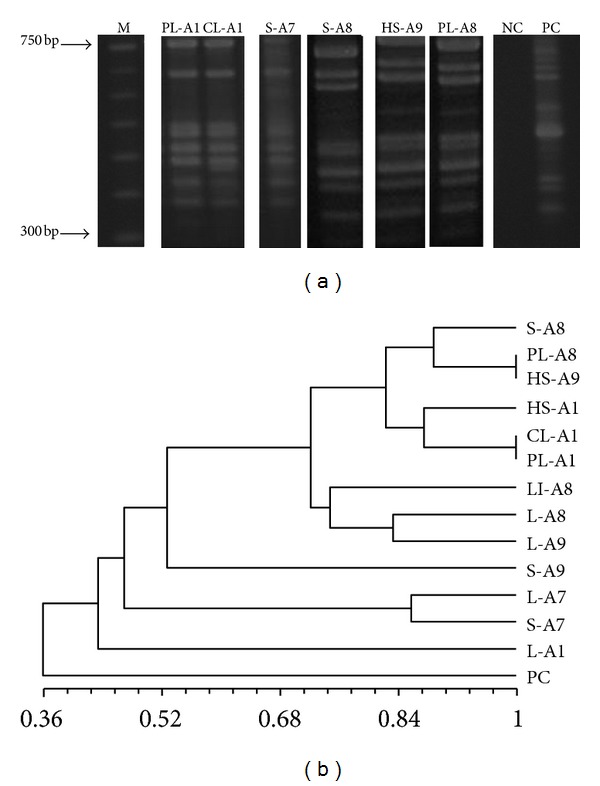
(a) Agarose gel electrophoresis showing similar and divergent representative genetic profiles generated by LSSP-PCR from parasites that disseminate to different anatomical sites. (b) Phenetic analysis using the Jaccard coefficient of similarity and UPGMA algorithm. M: 100 bp DNA ladder marker; NC: negative control; PC: positive control (*L. (V.) braziliensis* reference strain MHOM/BR/1975/M2903). S-A8: spleen fragment from animal 8; PL-A8: popliteal lymph node fragment from animal 8; HS-A9: healthy skin fragment from animal 9; HS-A1: healthy skin fragment from animal 1; CL-A1: cervical lymph node fragment from animal 1; PL-A1: popliteal lymph node fragment from animal 1; LI-A8: liver fragment from animal 8; L-A8: lesion fragment from animal 8; L-A9: lesion fragment from animal 9; S-A9: spleen fragment from animal 9; L-A7: lesion fragment from animal 7; S-A7: spleen fragment from animal 7; L-A1 lesion fragment from animal 1.

**Figure 3:**
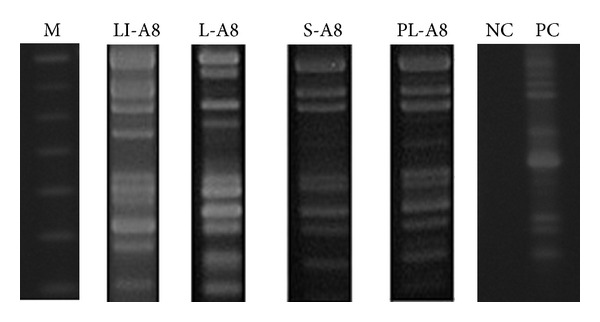
Agarose gel electrophoresis showing genetic profiles generated by LSSP-PCR for cutaneous lesion and internal organs from the same animal. M: 100 bp DNA ladder marker; LI-A8: liver fragment from animal 8; L-A8: cutaneous lesion fragment from animal 8; S-A8: spleen fragment from animal 8; PL-8: popliteal lymph node fragment from animal 8; NC: negative control; PC: positive control (*L. (V.) braziliensis *reference strain MHOM/BR/1975/M2903).

The genetic profiles generated by LSSP-PCR were submitted to phenetic analyses, and the band matrices were transformed into dendrograms. The first generated dendrogram (Figure 1) has grouped populations present in the cutaneous lesions of eight dogs. *L. (V.) braziliensis* populations with no dissemination attributes were found to be genetically similar, showing Sm coefficients of similarity varying from 0.9 to 1.00, and they were placed in a separate cluster. On the other hand, populations that showed spreading to internal organs have revealed more polymorphic genetic profiles with the coefficient of similarity ranging from 0.36 to 0.9. Six distinct genetic profiles were identified in the cutaneous lesions. The second dendrogram (Figure 2) has grouped disseminated populations that were found in different internal organs, cutaneous lesions, and intact skin. Despite the observed heterogeneity, populations with identical genetic profiles present in the popliteal (PL-A1) and in cervical (CL-A1) lymph nodes of the same animal were distinguished. Moreover, different genetic profiles in the same anatomical site but in different dogs (S-A7 and S-A8) and identical profiles in distinct sites in different animals (HS-A9 and PL-A8) were also observed. Eleven genetically dissimilar *L. (V.) braziliensis* populations were found in this cohort. However, parasite populations presenting the same genetic profile of that detected in cutaneous lesions were not observed in the internal organs of the same animal (Figure 3).

## 4. Discussion

In this study, using a polymorphic molecular marker, we have demonstrated the genetic heterogeneity in kDNA minicircles fragments from *L. (V.) braziliensis* strains, which have been shown to be composed of populations that differ in their propensity to disseminate to internal organs. The reproducibility of the genetic profile allows us to affirm that the observed heterogeneity was not due to the amount of amplified product loaded on the gel or artifacts.

In endemic areas where different *Leishmania *species or variants are transmitted, the disease can be the result of a heterogeneous infective inoculum, probably due to the accumulation of multiple independent infections [10]. In fact, the presence of distinct parasite populations circulating in endemic areas of CL is known, and the multiclonal origin of *Leishmania* strains has already been shown [11].

Studies on the ability of dissemination and tissue tropism of *L. (V.) braziliensis* in dogs are scarce in the literature. According to the results presented in this study, the ability of the pathogen to disseminate to internal organs or even to healthy skin seems to be related to the presence of specific populations of *L. (V.) braziliensis*. It was observed that the parasite populations that spread to other anatomic sites are genetically distinct from those in which the parasite was restricted to the skin lesion.

Whether the observed genetic polymorphisms reflect genetic plasticity [12] or indicate that the strains detected in the cutaneous lesions are composed of more than one population cannot be unequivocally ascertained from our data. Further clonal analysis of such strains will perhaps solve this question. On the other hand, such variation might reflect a shift in specific classes of minicircles [13] or alternatively an endogenous generation of new polymorphisms [14]. Since the spreading of a specific genetic profile of parasite recovered from cutaneous lesions was not noted in the internal organs of the same animal, the endogenous generation of polymorphisms is a plausible explanation.

The genetic polymorphisms observed in these populations can also be regarded as a way of conferring selective advance to the parasite. This argument is supported by the recent report on the presence of certain genetic patterns of *L. (V.) braziliensis* in HIV-positive patients [15]. In addition, another study [16] on the variability of *L. (L.) infantum* in humans and dogs has addressed the possibility that the immune system can select virulent over avirulent *Leishmania* populations. The genetic diversity of the circulating *L. (V.) braziliensis* strains can make the association between a specific genetic profile and tissue tropism very difficult. Although identical genetic profiles in different anatomical sites of the same animal (PL-A1 and CL-A1) and in different animals (HS-A9 and PL-A8) have been observed in the present study, the spreading of a specific parasite genetic profile from cutaneous lesions to internal organs was not evident. Results of a previous study corroborate this finding that no association was observed between the genetic profile and the clinical condition of the patients [17]. However, in contrast, genetically divergent profiles were detected in lesions from patients biopsied at different times within a period of one year [18].

To our knowledge, this is the first study reporting the genetic heterogeneity of *L. (V.) braziliensis* populations present in anatomical sites other than the cutaneous lesions in naturally infected dogs from endemic areas of Rio de Janeiro, Brazil. The finding of *L. (V.) braziliensis* strains that remain restricted to cutaneous lesions and other strains that disseminate to internal organs showing distinct genetic profiles, probably as a consequence of kDNA mutation, suggests the possibility that the dispersal parasites are viable. Reports from our group and others have showed that persistence of *Leishmania (Viannia) *parasites is probably the rule instead of an exception. Reactivation of cutaneous lesions [10] or the PCR detection of *L. (V.) braziliensis* DNA, followed by the isolation of viable parasites 11 years after the clinical cure of CL [19], reinforces this affirmation.Further studies with a larger number of clinical samples from naturally infected dogs are needed in order to draw correlations between genetic profiles and pathogenicity.

## 5. Conclusion

Our results support the idea that infection in dogs can be manifested by dissemination and tissue tropism of genetically distinct populations of *L. (V.) braziliensis*.

## Figures and Tables

**Table 1:** Geographical origin, localization of lesions, and IFAT titers of the animals studied.

Animals	Origin	Lesion	Titers
A1	Campo Grande	Scrotum	1 : 160
A2	Mangaratiba	Ear	1 : 160
A3	Campo Grande	Scrotum	1 : 160
A4	Campo Grande	Muzzle	1 : 80
A5	Miguel Pereira	Muzzle	1 : 80
A6	Jacarepaguá	Scrotum	1 : 40
A7	Maricá	Ear	1 : 80
A8	Rio de Janeiro	Ear	1 : 40
A9	Ilha Grande	Muzzle	1 : 640

**Table 2:** PCR results in the different tissue fragments from nine dogs studied.

Dog	Lesion	Health skin	Spleen	Liver	Popliteal lymph node	Cervical lymph node
A1	+	+	+	−	+	+
A2	+	+	−	+	+	+
A3	+	−	−	−	−	−
A4	+	−	−	−	−	−
A5	+	−	−	−	−	−
A6	+	−	−	−	−	−
A7	+	−	+	−	ND	−
A8	+	−	+	+	+	−
A9	+	+	+	−	ND	−

ND: not done; +: positive results; −: negative results.

## References

[1] Desjeux P (2004). Leishmaniasis: current situation and new perspectives. *Comparative Immunology, Microbiology and Infectious Diseases*.

[2] Aguilar CM, Rangel EF, Grimaldi Filho G, Momem H (1987). Human, canine and equine leishmaniasis caused by *Leishmania braziliensis braziliensis* in an endemic area in the State of Rio de Janeiro. *Memorias do Instituto Oswaldo Cruz*.

[3] Barbosa-Santos EG, Marzochi MC, Urtado W, Queirós F, Chicarino J, Pacheco RS (1994). Leishmaniasis disseminated by *Leishmania braziliensis* in a mare (*Equus cabalus*) immunotherapy and chemotherapy assays. *Memorias do Instituto Oswaldo Cruz*.

[4] Marzochi MCA, Marzochi KB (1994). Tegumentary and visceral leishmaniasis in Brazil—emerging anthropozoonosis and possibilities for their control. *Cadernos de Saúde Pública*.

[5] Velasquez LG, Membrive N, Membrive U (2005). PCR in the investigation of canine American tegumentary leishmaniasis in northwestern Paraná State, Brazil. *Cadernos de Saude Publica*.

[6] Ministerio da Saúde (2006). *Manual de vigilância e controle da Leishmaniose Visceral*.

[7] Oliveira FS, Pirmez C, Pires MQ, Brazil RP, Pacheco RS (2005). PCR-based diagnosis for detection of *Leishmania* in skin and blood of rodents from an endemic area of cutaneous and visceral leishmaniasis in Brazil. *Veterinary Parasitology*.

[8] de Bruijn MHL, Barker DC (1992). Diagnosis of new world leishmaniasis: specific detection of species of the *Leishmania braziliensis* complex by amplification of kinetoplast DNA. *Acta Tropica*.

[9] Sneath PHA, Sokal RR (1962). Numerical taxonomy. *Nature*.

[10] Saravia NG, Weigle K, Segura I (1990). Recurrent lesions in human *Leishmania braziliensis* infection—reactivation or reinfection?. *The Lancet*.

[11] Pacheco RS, Grimaldi G, Momen H, Morel CM (1990). Population heterogeneity among clones of new world *Leishmania* species. *Parasitology*.

[12] Pacheco RS, Brito CM (1999). Reflections on the population dynamics of *Trypanosoma cruzi*: heterogeneity versus plasticity. *Memorias do Instituto Oswaldo Cruz*.

[13] Borst P (1991). Why kinetoplast DNA networks?. *Trends in Genetics*.

[14] Pacheco RS, Martinez JE, Valderrama L, Momen H, Saravia NG (1995). Genotypic polymorphisms in experimental metastatic dermal leishmaniasis. *Molecular and Biochemical Parasitology*.

[15] Oliveira FS, Valete-Rosalino CM, Schubach AO, Madeira MF, Pacheco RS (2012). Genetic polymorphism in *Leishmania (Viannia) braziliensis* detected in mucosal leishmaniasis of HIV-infected and non-HIV-infected patients. *Transactions of the Royal Society of Tropical Medicine and Hygiene*.

[16] Jimenez M, Ferrer-Dufol M, Canavate C (1995). Variability of *Leishmania (Leishmania)* infantum among stocks from immunocompromised, immunocompetent patients and dogs in Spain. *FEMS Microbiology Letters*.

[17] Baptista C, Schubach AO, Madeira MF (2009). *Leishmania (Viannia) braziliensis* genotypes identified in lesions of patients with atypical or typical manifestations of tegumentary leishmaniasis: evaluation by two molecular markers. *Experimental Parasitology*.

[18] de Oliveira FS, Valete-Rosalino CM, Schubach AO, Pacheco RS (2010). kDNA minicircle signatures of *Leishmania (Viannia) braziliensis* in oral and nasal mucosa from mucosal leishmaniasis patients. *Diagnostic Microbiology and Infectious Disease*.

[19] Schubach A, Marzochi MCA, Cuzzi-Maya T (1998). Cutaneous scars in American tegumentary leishmaniasis patients: a site of *Leishmania (Viannia) braziliensis* persistence and viability eleven years after antimonial therapy and clinical cure. *American Journal of Tropical Medicine and Hygiene*.

